# A Comparative Study of Outcomes of Submucosal Diathermy Versus Inferior Turbinoplasty in Patients With Deviated Nasal Septum With Inferior Turbinate Hypertrophy

**DOI:** 10.7759/cureus.64301

**Published:** 2024-07-11

**Authors:** Vinod Shinde, Aishwarya Kothari, Mayur Ingale, Anuja Satav

**Affiliations:** 1 Department of Otorhinolaryngology, Dr. D. Y. Patil Medical College, Hospital, and Research Centre, Dr. D. Y. Patil Vidyapeeth, Pune, IND

**Keywords:** snot-22 questionnaire, inferior turbinoplasty, submucosal diathermy, inferior turbinate hypertrophy, deviated nasal septum

## Abstract

Introduction

Nasal obstruction due to deviated nasal septum (DNS) and inferior turbinate hypertrophy (ITH) is a common problem necessitating surgical intervention. Submucosal diathermy (SMD) and inferior turbinoplasty (IT) are two commonly performed procedures aimed at improving nasal patency.

Methods

A prospective comparative study was conducted on 56 patients with DNS and inferior turbinate hypertrophy, divided into SMD and inferior turbinoplasty groups. Preoperative and postoperative assessments included symptom assessment using the 22-item Sinonasal Outcome Test (SNOT-22) questionnaire.

Results

Both procedures led to significant improvements in nasal symptoms and quality of life. Inferior turbinoplasty showed slightly better outcomes in symptom improvement compared to submucosal diathermy.

Conclusions

Inferior turbinoplasty appears to offer slightly better outcomes in improving nasal symptoms compared to SMD in patients with DNS and inferior turbinate hypertrophy. However, both procedures are effective and safe options for surgical management. Individualized treatment decisions should consider patient preferences and surgeon expertise.

## Introduction

The nasal cavity serves as the primary entrance for inspired air into the respiratory system and plays a crucial role in humidifying, warming, and filtering the air [1]. The anatomy of the nasal cavity is complex, consisting of various structures, including the nasal septum and turbinates, which are integral for optimal nasal function [2].

Deviated nasal septum (DNS) and inferior turbinate hypertrophy (ITH) represent two common anatomical abnormalities that can contribute to nasal airway obstruction and impair nasal function [3]. DNS refers to a displacement of the nasal septum from its midline position, whereas ITH refers to the enlargement of the inferior turbinates, typically due to mucosal hypertrophy. The turbinate thickens mostly owing to its anatomical nature but also as a result of nasal septal deviation or rhinitis. Both conditions can independently contribute to nasal obstruction and are frequently encountered together, exacerbating symptoms and complicating treatment strategies [2].

Nasal obstruction can significantly impair the quality of life by affecting sleep, daily activities, and overall well-being [1]. Surgical management of DNS and ITH aims to improve nasal airflow, alleviate symptoms of obstruction, and enhance quality of life. Among the surgical techniques available, submucosal diathermy (SMD) and inferior turbinoplasty (IT) have gained popularity due to their effectiveness, safety, and relatively low morbidity [4].

Submucosal diathermy (SMD) aims to reduce the volume of the hypertrophied turbinate tissue by delivering thermal energy to the submucosal layer, leading to tissue contraction and improvement in nasal airflow [5-7]. During SMD, a cautery device or radiofrequency probe is inserted into the inferior turbinate, and energy is delivered to the submucosal tissue. This results in controlled tissue ablation and fibrosis, leading to long-term reduction in turbinate size and improved nasal patency [8].

Inferior turbinoplasty (IT) encompasses various procedures aimed at reducing turbinate bulk, including partial resection, submucosal resection, and outfracture techniques [4]. Partial turbinectomy involves the surgical excision of a portion of the inferior turbinate. Submucosal resection techniques involve the removal of hypertrophied turbinate tissue while preserving the overlying mucosa. Outfracture techniques involve fracturing the inferior turbinate bone to create additional space within the nasal cavity. Laser turbinoplasty utilizes lasers such as CO2 or diode lasers to selectively ablate turbinate tissue, resulting in the reduction of turbinate volume and improvement in nasal patency [8].

While both SMD and IT have demonstrated efficacy in improving nasal patency, limited comparative data exist regarding their respective outcomes in patients with DNS and ITH. Understanding the comparative effectiveness of these interventions is essential for guiding treatment decisions and optimizing patient care. By evaluating and comparing the outcomes of SMD and IT, this study seeks to provide valuable insights into the management of DNS and ITH.

## Materials and methods

The study was an outcome-based comparative study conducted at the department of ENT in a tertiary care hospital in western Maharashtra from October 2022 to March 2024. The study focused on patients who attended the ENT OPD. Ethical approval was obtained from the Institutional Ethics Committee of Dr. D. Y. Patil Vidyapeeth, Pune (approval number IESC/PGS/2022/121), before commencing the study. Written informed consent was taken from the patients participating in the study.

Patients of the age group 18-60 years with deviated nasal septum having inferior turbinate hypertrophy not responding to medical therapy for six weeks were included. Based on endoscopic findings and radiological findings, patients with a nasal polyp, a nasal mass, concha bullosa, or deviated nasal septum without inferior turbinate hypertrophy were excluded.

A total of 56 clinically suspected patients with deviated nasal septum having inferior turbinate hypertrophy, as a result of nasal septal deviation, were enrolled in the study, which were divided into two groups of 28 each. Those in group A were subjected to inferior turbinoplasty. At the same time, patients in group B were subjected to submucosal diathermy.

**Figure 1:**
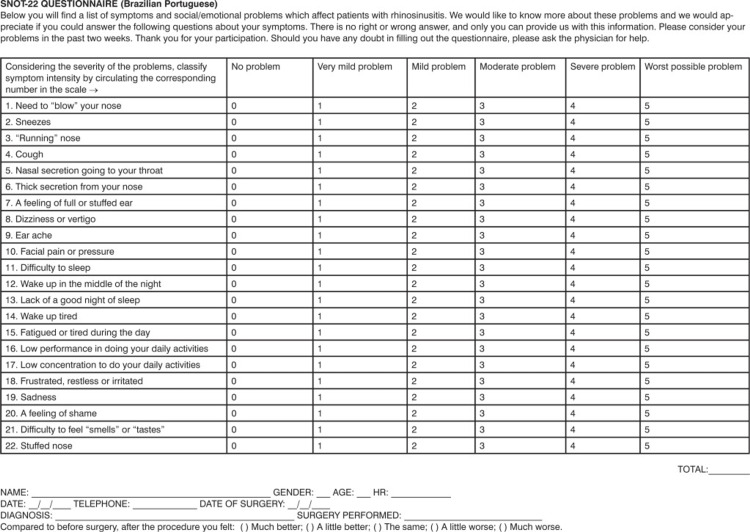
22-item Sinonasal Outcome Test questionnaire SNOT-22: Sinonasal Outcome Test

Prior to surgical intervention, the patients were asked to select the symptoms that were present in them out of the 22 symptoms present in the Sinonasal Outcome Test (SNOT-22) questionnaire (Figure 1), which had been used in a study conducted by Husain et al. [9].

The same was done postoperatively after three weeks, and overall symptom outcomes were compared.

Data analyses were done using SPSS version 27.0 (IBM SPSS Statistics, Armonk, NY). Categorical variables were displayed as frequency and percentage, while numerical data were presented as mean ± standard deviation (SD). Correlation analysis was done using Pearson's correlation test. The p-value was considered significant at <0.05.

## Results

In our study of 56 individuals (34 males and 22 females), the mean age was 26.03 ± 8.08 years with a range of 19-49 years. The mean age of the 28 patients in SMD (18 males and 10 females) was 27.46 ± 9.44 years, and the mean age of the 28 individuals in IT (16 males and 12 females) was 25.14 ± 6.22 years. These findings indicate that there was no significant difference in the mean age between the two groups, with both averaging around 26 years.

In the 18-20 years age group, there were nine patients each for both procedures. The 21-30 years age group had the highest number of patients, with 14 undergoing inferior turbinoplasty and nine undergoing submucosal diathermy. The 31-40 years age group included five for SMD and four patients for IT. In the 41-50 years age group, five patients underwent SMD, while one underwent IT (Table 1).

**Table 1:** Age-wise distribution of patients

Age group (years)	Inferior turbinoplasty	Submucosal diathermy
18-20	9	9
21-30	14	9
31-40	4	5
41-50	1	5
Total	28	28

Table 2 illustrates the comparison of preoperative symptoms and postoperative symptoms after three weeks for both submucosal diathermy and inferior turbinoplasty.

**Table 2:** Comparison of preoperative symptoms and postoperative symptoms after three weeks for submucosal diathermy and inferior turbinoplasty

Symptoms	Submucosal diathermy (preoperative)	Submucosal diathermy (postoperative)	Inferior turbinoplasty (preoperative)	Inferior turbinoplasty (postoperative)
Nasal blockage	25 (89%)	5 (18%)	26 (93%)	4 (14%)
Runny nose	22 (79%)	6 (21%)	21 (75%)	5 (18%)
Sneezing	18 (64%)	8 (29%)	19 (68%)	7 (25%)
Thick nasal discharge	15 (54%)	4 (14%)	14 (50%)	3 (11%)
Ear fullness	10 (36%)	3 (11%)	11 (39%)	2 (7%)
Dizziness	8 (29%)	2 (7%)	7 (25%)	1 (4%)
Decreased sense of taste and smell	12 (43%)	6 (21%)	13 (46%)	5 (18%)
Lack of good sleep	20 (71%)	7 (25%)	21 (75%)	6 (21%)
Fatigue	19 (68%)	6 (21%)	20 (71%)	5 (18%)
Reduced productivity	16 (57%)	5 (18%)	15 (54%)	4 (14%)
Reduced concentration	17 (61%)	4 (14%)	18 (64%)	3 (11%)
Need to blow nose	23 (82%)	7 (25%)	24 (86%)	6 (21%)
Cough	14 (50%)	4 (14%)	13 (46%)	3 (11%)
Post-nasal drip	20 (71%)	6 (21%)	21 (75%)	5 (18%)
Ear pain	9 (32%)	3 (11%)	10 (36%)	2 (7%)
Facial pain/pressure	11 (39%)	4 (14%)	12 (43%)	3 (11%)
Difficulty falling asleep	17 (61%)	5 (18%)	18 (64%)	4 (14%)
Waking up at night	15 (54%)	6 (21%)	16 (57%)	5 (18%)
Waking up tired	18 (64%)	5 (18%)	19 (68%)	4 (14%)
Frustration/restlessness/irritability	21 (75%)	7 (25%)	22 (79%)	6 (21%)
Sadness	13 (46%)	4 (14%)	12 (43%)	3 (11%)
Embarrassment	10 (36%)	3 (11%)	9 (32%)	2 (7%)

Preoperatively, the mean frequency of symptoms was 16.05 ± 4.69 for SMD and 16.41 ± 5.04 for IT. Postoperatively, after three weeks, the SMD showed a mean frequency of 5.00 ± 1.54 and IT showed an even lower mean frequency of 4.00 ± 1.54. The test of significance confirms these improvements, with SMD achieving a p-value of 0.0051 and IT achieving a more significant p-value of 0.0009, indicating that both treatments are effective, with IT demonstrating superior symptom reduction (Table 3).

**Table 3:** Comparison of preoperative and postoperative symptoms after three weeks with test of significance SD: standard deviation

Category	Submucosal diathermy (mean ± SD)	Inferior turbinoplasty (mean ± SD)
Preoperative overall	16.05 ± 4.69	16.41 ± 5.04
Postoperative overall	5.00 ± 1.54	4.00 ± 1.54
Test of significance	p = 0.0051	p = 0.0009

## Discussion

Patients with deviated nasal septum (DNS) often experience nasal obstruction, which can significantly impact their quality of life. Inferior turbinate hypertrophy (ITH) is a common coexisting condition that exacerbates symptoms. Surgical interventions, such as SMD and IT, are often necessary for symptom relief. Previous studies have shown variable outcomes and complication rates for these procedures [10,11].

The demographics of our study included 56 patients with a mean age of 26.03 ± 8.08 years, a median age of 24 years, a mode age of 19 years, and an age range from 19 to 49 years. In comparison, Taneja et al. [12] studied 80 patients divided into four groups of 20 each. Gomaa et al. [13] involved 50 patients of various ages and sexes, divided into two groups of 25 each. Datta et al. [14] focused on 60 patients with allergic rhinitis, randomized into two groups of 30 each.

The clinical presentation in our study included rhinological symptoms, ear and facial symptoms, sleep function, and psychological issues. In comparison, Taneja et al. [12] focused on nasal obstruction due to inferior turbinate hypertrophy. Gomaa et al. [13] studied chronic nasal obstruction due to inferior turbinate hypertrophy unresponsive to medical treatment. Datta et al. [14] reported severe nasal obstruction in 75% of patients and moderate obstruction in 25%.

Our study outcomes reveal significant postoperative symptom reductions in our study, with submucosal diathermy (SMD) reducing the mean frequency of symptoms from 16.05 ± 4.69 to 5.00 ± 1.54 (p = 0.0051) and inferior turbinoplasty from 16.41 ± 5.04 to 4.00 ± 1.54 (p = 0.0009). Taneja et al. [12] reported significant improvement in nasal obstruction and quality of life, with maintenance of mucociliary function across all groups. Gomaa et al. [13] found SMD to be superior to partial surgical inferior turbinectomy (PSIT) in reducing postoperative nasal pain and intranasal crusting, although both techniques were equally effective in alleviating nasal obstruction and ensuring tissue healing over the long term. Datta et al. [14] concluded that both procedures effectively reduced nasal obstruction and allergic rhinitis symptoms, with SMD showing superior results at the three-month follow-up and partial inferior turbinectomy (PIT) being more effective at six months. They recommended SMD for its less invasive nature and fewer postoperative complications.

Al-Baldawi et al. [15] compared partial turbinectomy with submucosal diathermy and found that postoperative nasal obstruction improvement was seen in 82.5% of patients who underwent submucosal diathermy and 97.5% of patients who underwent turbinectomy. Our study similarly showed significant improvement in nasal symptoms after both submucosal diathermy and inferior turbinoplasty, corroborating the efficacy of these procedures in relieving nasal obstruction.

The results of our study indicate that both SMD and ITP are effective surgical options for patients with DNS and inferior turbinate hypertrophy. Both procedures resulted in significant improvements in preoperative symptoms and quality of life. The choice between SMD and ITP should be based on individual patient factors, surgeon experience, and patient preferences.

For patients with mild to moderate symptoms and a preference for less invasive procedures, SMD may be a suitable option. On the other hand, patients with more severe symptoms and a desire for more extensive tissue removal may benefit from ITP. This may be attributed to the more extensive tissue removal achieved with ITP, leading to better symptom relief, particularly for symptoms such as runny nose, sneezing, and thick nasal discharge.

However, it is essential to consider other factors such as surgical complexity, procedure time, and postoperative recovery.

Study limitations

This was a single-center study with a relatively small sample size, which may limit the generalizability of the findings. Future studies with larger sample sizes and multicenter designs are needed to confirm our results. The follow-up period in our study was relatively short term, and longer-term outcomes beyond the follow-up period were not assessed. Other surgical techniques for inferior turbinate reduction, such as radiofrequency ablation and laser-assisted turbinoplasty, were not included in our study.

## Conclusions

This study compared submucosal diathermy (SMD) and inferior turbinoplasty (IT) in patients with deviated nasal septum (DNS) and inferior turbinate hypertrophy (ITH), finding that both improved nasal patency and symptoms, with IT showing greater postoperative symptom reduction. These findings indicate that, while both procedures are effective, IT offers superior symptom relief. However, the choice between SMD and IT should be guided by individual patient characteristics, surgeon expertise, and patient preferences. Patients seeking a less invasive option with faster recovery might prefer SMD, while those requiring more extensive tissue removal for severe symptoms may benefit more from IT. Further research with larger, multicenter studies and longer follow-up periods is recommended to validate these results and refine treatment protocols.
